# Improved participants’ understanding in a healthy volunteer study using the SIDCER informed consent form: a randomized-controlled study

**DOI:** 10.1007/s00228-015-2000-2

**Published:** 2015-12-29

**Authors:** Nut Koonrungsesomboon, Supanimit Teekachunhatean, Nutthiya Hanprasertpong, Junjira Laothavorn, Kesara Na-Bangchang, Juntra Karbwang

**Affiliations:** Department of Clinical Product Development, Institute of Tropical Medicine, Nagasaki University, 1-12-4 Sakamoto, Nagasaki, 852-8523 Japan; Leading Program, Graduate School of Biomedical Sciences, Nagasaki University, Nagasaki, Japan; Department of Pharmacology, Faculty of Medicine, Chiang Mai University, Chiang Mai, 50200 Thailand; Chulabhorn International College of Medicine, Thammasat University, Pathum Thani, 12121 Thailand

**Keywords:** Informed consent, Clinical research, Clinical study, Healthy subjects, Healthy volunteers, Understanding

## Abstract

**Purpose:**

This study aimed to evaluate the applicability of the principles and informed consent form (ICF) template proposed by the Strategic Initiative for Developing Capacity in Ethical Review (SIDCER) in a clinical pharmacokinetic study by comparing the volunteers’ understanding of the enhanced ICF (developed based on the SIDCER methodology) and the conventional ICF (which was previously approved by local Ethics Committee and used in the clinical study).

**Methods:**

A total of 550 volunteers were randomly assigned to read either the enhanced ICF or the conventional ICF (1:1) in a mock informed consent approach and subsequently performed the post-test questionnaire. The primary endpoint was the proportion of the participants who had the post-test score of ≥80 %; the secondary endpoints were the total score of the post-test, the score of the categorized ICF elements, and time spent for participation.

**Results:**

The proportion of the participants in the enhanced ICF group who achieved the primary endpoint was significantly higher than the conventional ICF group (82.2 % vs. 60.4 %, *p* < 0.001). The participants in the enhanced ICF group obtained higher scores and spent less time in reading the given ICF and answering the post-test than those in the conventional ICF group (total score 19/21 vs. 18/21, *p* < 0.001; time spent 20 min vs. 25 min, *p* < 0.001).

**Conclusion:**

The enhanced ICF improved the understanding of the participants in this study. This demonstrates the applicability of the SIDCER ICF principles and its template in the development of an enhanced ICF for improving the quality of ICFs and subjects’ understanding in clinical research.

Trial registration: TCTR20140727001

## Introduction

The subjects’ limited understanding of trial information has been a continuing source of concern especially in non-therapeutic studies involving healthy volunteers. After the incident of the TGN1412 study, there has been an increasing concern regarding the validity of informed consent in healthy volunteers in phase I studies. All healthy volunteers who received an intervention in the TGN1412 study ended up in the intensive care unit [1]. Investigations of how much the volunteers understood the risks involved in the study show that the informed consent form (ICF) was extensively long (13 pages), lacked many essential elements, and made liberal use of complex language [2]. The volunteers did not have a real understanding of the risks that would be involved in the study or recognize the risks when participating in the study [3]. This study highlights concerns about the safety of healthy volunteers in clinical trials. Comprehension of research subjects, particularly concerning their rights and the risks involved in clinical research, has become a matter of paramount importance [4, 5]. Literature reviews demonstrate limited understanding of fundamental trial aspects among healthy research volunteers, even experienced ones who were enrolled in the long-duration clinical trials [5–7]. Some of them could not even name the drug they were taking or recall any side effects of that drug [6]. These cases call into question the quality and validity of informed consent currently being practiced in clinical research.

An ICF is a required document in clinical studies, which provides information of a particular study to potential research subjects and documents an individual subject’s voluntary consent to participate in research. To date, the quality of ICFs used in clinical studies is still problematic due mainly to extensive length [8–10], chaotic structure, and overly technical language [11, 12]. These potentially reduce the subject’s ability to thoroughly read and understand the information provided therein [13], thus compromising the ethical principle of respect for persons [14]. Additionally, information essential for a subject’s decision-making is sometimes missing in many ICFs [15]. Improvement of the quality of ICFs in clinical trials is thus necessary for ethical research conduct. For decades, ICFs have been extensively refined and two systematic reviews demonstrate the effectiveness of an enhanced or simplified ICF in improving subjects’ understanding in clinical research [16, 17].

Recently, the Strategic Initiative for Developing Capacity in Ethical Review (SIDCER), an organization launched by the Special Programme for Tropical Disease Research, World Health Organization in 2001, has proposed the SIDCER ICF principles and template for ICF development. The concept and structure of the SIDCER ICF have been refined and finalized in collaboration with researchers and Ethics Committee members mainly from the Forum of Ethical Review Committees in the Asian and Western Pacific region (FERCAP). In short, the SIDCER ICF consists of three principles: (1) completeness of the mandatory ICF content required by international ethics guidelines and regulations, (2) concise information provision which is relevant to a subject’s decision-making and local context, and (3) use of simplified means to effectively convey relevant information to the target population. The SIDCER ICF template for clinical trials has been designed to guide the development of an ICF based on the SIDCER ICF principles (presented in the 14th FERCAP Conference, Tagaytay, Philippines, 2014). Validation of the SIDCER ICF’s applicability and effectiveness in a variety of clinical studies with different designs is needed. Although the concept is universal, the application and validation must be done in a local language as it plays a major role in the comprehension of research subjects in a reference setting and context.

The present study is an empirical study to test the SIDCER ICF’s applicability and effectiveness in Thai and to evaluate the understanding of Thai volunteers. The SIDCER ICF methodology was applied to a clinical pharmacokinetic (PK) drug-drug interaction (DDI) study involving healthy subjects, and the local volunteers’ understanding between the enhanced (SIDCER) ICF and the conventional ICF was compared using a randomized-controlled design.

## Methods

This study was an open-label, randomized-controlled study of the two different ICF interventions (1:1), i.e., the enhanced ICF and the conventional ICF, using a post-test questionnaire as an assessment tool. The clinical study protocol of the PK DDI study entitled “Effect of curcuminoids on the blood level of digoxin in healthy male volunteers” (Trial Registration Number: ChiCTR-TTRCC-14004645) was used to be the core information for ICF development.

### The enhanced informed consent form

Following the SIDCER ICF principles and template, the enhanced ICF was developed (in Thai) based on the abovementioned clinical study protocol. In close observation of the first SIDCER ICF principle, the study protocol was reviewed and the essential information in regard to compliance with internationally recognized regulatory ICF requirements are selected and present in the enhanced ICF. To limit the contents and length of the enhanced form in accordance with the second SIDCER ICF principle, the information in the study protocol was carefully analyzed and summarized, and only relevant information to a subject’s decision-making on research matters was used for ICF development. The refined information was then transformed into a narrative and illustrative format and appropriately inserted into the SIDCER ICF template (where the third SIDCER ICF principle is applied) using local lay language with reference to local context.

A four-page enhanced ICF containing 21 required elements was successfully developed (Table 1). It was then reviewed by the principal investigator of the PK DDI study (NH) to ensure the accuracy of information and by two independent local laypersons to ensure the use of the simplest terms with reference to local context.

**Table 1:** The enhanced ICF and the conventional ICF

	Enhanced ICF	Conventional ICF
Number of pages	4	7
Number of required elements provided	21	15
General items		
Recognition that this is research	✓	✓
Subject’s responsibility	✓	✓
Confidentiality of records	✓	✓
Who can access the data	✓	Not stated
Research contact person(s)	✓	✓
Rights of the subject		
Right to refuse	✓	✓
Right to withdraw	✓	✓
Consequences of withdrawal	✓	Not stated
Right to receive new relevant information	✓	Not stated
Scientific aspects		
Subject eligibility	✓	✓
Number of subjects required	✓	✓
Purpose of the study	✓	✓
Trial treatment	✓	✓
Trial procedures	✓	✓
Duration of the subject’s participation	✓	Not stated
Ethical aspects		
Foreseeable risks	✓	✓
Expected direct and/or indirect benefits	✓	Not clear^a^
Participant termination criteria	✓	Not stated
Prorated payment for participation	✓	✓
Anticipated expenses	✓	✓
Compensation for injury	✓	✓

^a^“No direct benefit from study participation” was not stated

### The conventional informed consent form

The original ICF (in Thai) used in the abovementioned PK DDI study was considered as a conventional ICF in the present ICF study. This ICF was previously approved by the Research Ethics Committee of the Faculty of Medicine, Chiang Mai University, 2 years prior to the development of the SIDCER ICF, and it was used in the real informed consent process of that study. It had seven pages containing 15 required elements (Table 1).

### The post-test questionnaire

The post-test questionnaire (in Thai) consisted of 21 short case studies where each case study was designed to illustrate a common practical situation of one element required, followed by a question with three possible answers. It was used as an assessment tool to determine the volunteers’ understanding of each required element after they read a given ICF. All case studies and answer choices were also reviewed and corrected by the principal investigator of the PK DDI study (NH) and two independent laypersons.

### Study endpoints

The primary endpoint was the proportion of the participants who had the post-test score of more than 80 %. The secondary endpoints were the total score of the post-test, the score of each category of elements required (Table 1), and time spent for reading a given ICF and doing the post-test questionnaire.

### Sample size determination

The estimated sample size was calculated using G*power 3.1.9.2 for Windows. The *z*-test to detect the 10 % difference between two independent proportions of the primary endpoint (*p*_1_ = 0.85 and *p*_2_ = 0.75) was applied, with the precision and confidence level of 95 % (α = 0.05) and 80 % power (1 − β = 0.80), using continuity correction with allocation ratio of 1. A sample size of at least 269 subjects is adequate for each group.

### Study procedure

Volunteers (age > 18) who can read and write Thai were recruited from universities, colleges, cafeterias, hospitals, and markets in Chiang Mai, Thailand. Individuals who had no time to read the entire ICF and do the post-test questionnaire were excluded. All processes were done in an anonymous manner using a subject unique number. The study protocol and all related documents were approved by the Research Ethics Committee of the Faculty of Medicine, Chiang Mai University. All procedures performed in this study were in accordance with the ethical standards of the institutional and national research committee and with the 2013 Helsinki declaration. Written informed consent was obtained from all individual participants prior to the enrollment of the study. This study was registered as TCTR20140727001 via the Thai Clinical Trial Registry.

**Fig. 1:**
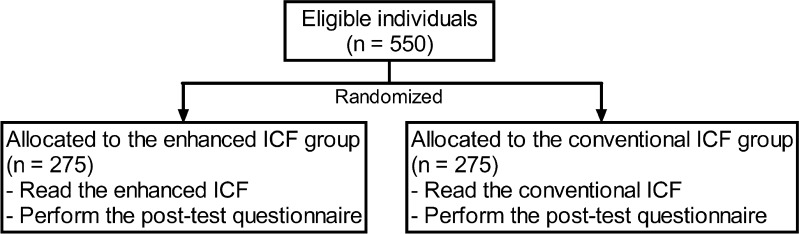
Study procedure

There were two main processes in this study: (1) the process of reading a given ICF and (2) the process of completing the post-test questionnaire. The participants were randomly assigned to read either the enhanced ICF or the conventional ICF (Fig. 1). After receiving an ICF (the enhanced ICF or the conventional ICF), individuals were given an unlimited time to read the given ICF. The post-test questionnaire was then distributed, and individuals were allowed to keep and consult the ICF while completing the questionnaire. If any question was unclear or no information was given by the ICF, individuals could mark down “no answer” or “unable to answer” in the questionnaire. The time spent on the two processes was recorded.

### Data analysis

Of the three possible answers in each question, there was only one correct answer, counting as a score of 1, making the total score 21. Incorrect answer, no answer or unable to answer in each question was counted as a score of 0. The chi-square test was applied for the comparison of the difference of nominal data between the two groups. The interval data were analyzed by nonparametric statistics and presented in a median (interquartile) since none of them were normally distributed. Subgroup analyses were performed by applying the chi-square test to determine the impact of genders (male and female), generations (generation Y, participants who were born between 1982 and 2000; generation X, participants who were born between 1965 and 1981; and baby boomers, participants who were born between 1946 and 1964) [18] and educational levels (level 1, high school or lower; level 2, bachelor degree or equivalent, diploma degree, or undergraduate level; and level 3, master/doctoral degree or equivalent, or graduate level) on the primary outcome. A statistical significance was set at α = 0.05 for all tests. Statistical analysis was performed using standard statistical software (SPSS version 22.0). Figures were generated using GraphPad Prism ver. 5.0.

## Results

A total of 550 participants, aged 18–67 years (median age = 24 years), were enrolled: 62 % were female; 66.9 % were under generation Y; and 76 % had educational level 2. Their demographic data are shown in Table 2.

**Table 2:** Demographic data of the participants (*n* = 550)

	Enhanced ICF (*n* = 275)	Conventional ICF (*n* = 275)
Gender		
Male	102	107
Female	173	168
Generation		
Generation Y	173	195
Generation X	72	58
Baby boomers	30	22
Educational level		
Level 1	44	43
Level 2	208	210
Level 3	23	22

Data represent the number of participants. Generation was divided into three subgroups: (1) generation Y includes participants who were born between 1982 and 2000; (2) generation X includes participants who were born between 1965 and 1981; and (3) baby boomers includes participants who were born between 1946 and 1964. Educational level was divided into three sub-levels: level 1 represents high school level or lower; level 2 represents bachelor degree or equivalent, diploma degree, or undergraduate level; and level 3 represents master/doctoral degree or equivalent, or graduate level.

**Fig. 2:**
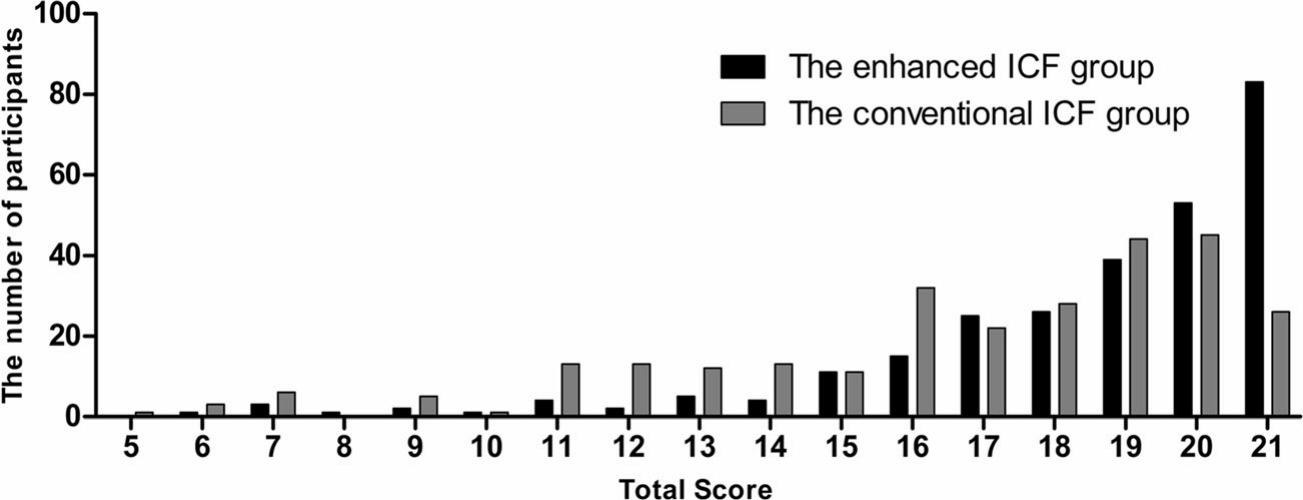
The distribution of the post-test score of the participants. *Black bars* represent the enhanced ICF group; *gray bars* represent the conventional ICF group

**Fig. 3:**
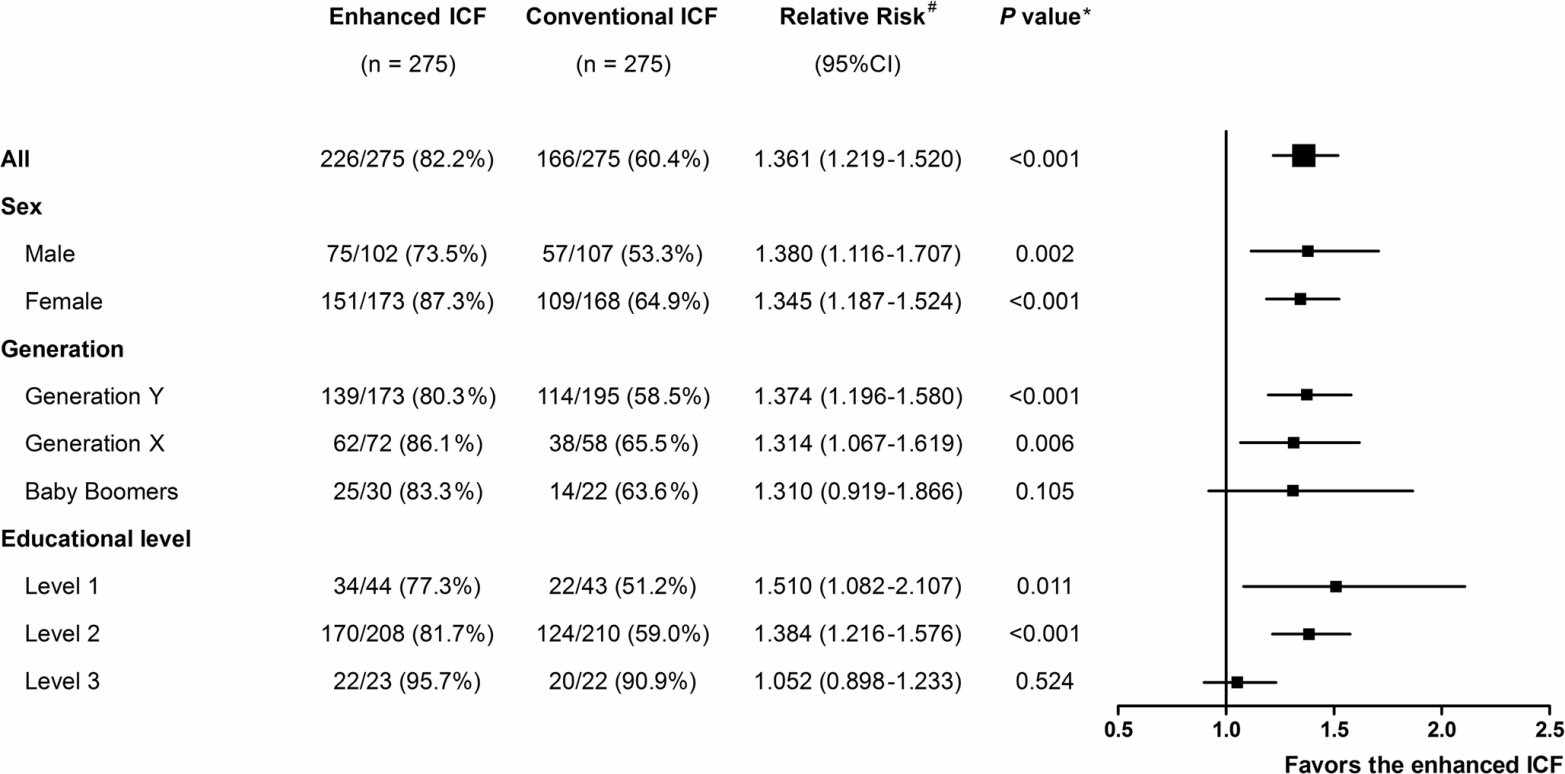
Proportions of the participants whose post-test score was satisfied according to the 80 % passing level (the primary endpoint). ^#^ Relative risk is the ratio derived from the proportion of the participants in the enhanced ICF group whose post-test scores were satisfied according to the 80 % passing level divided by that of the conventional ICF group. * Chi-square test

**Fig. 4:**
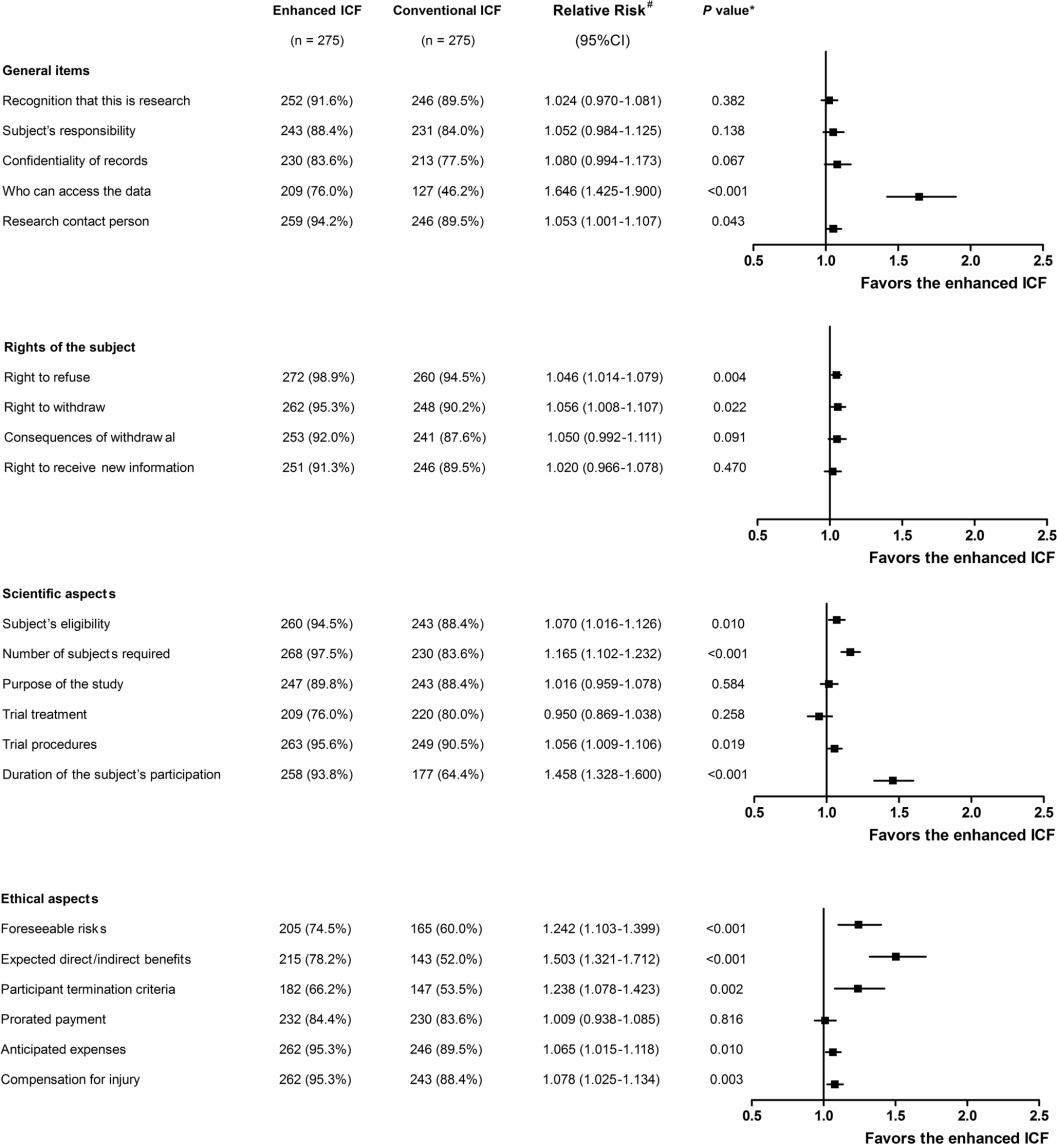
Proportions of the participants who correctly answered each item in the post-test questionnaire. ^#^ Relative risk is the ratio derived from the proportion of the participants in the enhanced ICF group who correctly answered each item in the post-test questionnaire divided by that of the conventional ICF group. * Chi-square test

The distribution of the post-test score of the participants in each group is shown in Fig. 2. Eighty-three participants (30.2 %) in the enhanced ICF group and 26 participants (9.5 %) in the conventional ICF group achieved the perfect score (RR 3.192, 95 % CI 2.124–4.798, *p* < 0.001). The proportion of the participants in the enhanced ICF group who achieved the primary endpoint was significantly higher than that in the conventional ICF group (82.2 % vs. 60.4 %, RR 1.361, 95 % CI 1.219–1.520, *p* < 0.001) (Fig. 3). Subgroup analyses by gender, generation, and educational level demonstrated that all but the participants in the baby boomers generation and participants who had educational level 3 favored the enhanced ICF (Fig. 3). The proportion of the participants in the enhanced ICF group who correctly answered each item in the post-test questionnaire was compared to that in the conventional ICF group: significant differences were seen in 13 out of 21 items (Fig. 4).

The assessment of the secondary endpoints showed that the total score of the post-test and the score of each category in the enhanced ICF group were significantly higher than those in the conventional ICF group (total score 19 vs. 18, *p* < 0.001; score of general items 5 vs. 4, *p* < 0.001; score of rights of the subject 4 vs. 4, *p* = 0.023; score of scientific aspects 6 vs. 5, *p* < 0.001; score of ethical aspects 5 vs. 4, *p* < 0.001). The time spent by the participants in the enhanced ICF group was significantly lesser than that in the other group (20 min vs. 25 min, *p* < 0.001).

## Discussion

The present study demonstrates the applicability of the SIDCER ICF and its template in the development of an enhanced ICF for the clinical PK trial and its effectiveness in improving the participants’ understanding of information related to the trial. Higher proportions of the participants who read the enhanced ICF obtained the satisfactory level of understanding at 80 % and perfect score in the post-test questionnaire than those who read the conventional ICF (82.2 % vs. 60.4 %, *p* < 0.001, and 30.2 % vs. 9.5 %, *p* < 0.001, respectively). The participants in the enhanced ICF group spent less time but achieved higher scores on the post-test when compare to those in the conventional ICF group (time spent 20 vs. 25, *p* < 0.001; total score 19 vs. 18, *p* < 0.001).

This study was the first study conducted to validate the applicability and effectiveness of the SIDCER ICF, using a randomized-controlled design. The results indicate that shortening the length of an ICF is possible while retaining all essential elements in a concise manner and that the SIDCER ICF template is applicable to a clinical PK study and its application is effective in this group of population. Since literature reviews demonstrate the mixed results of other ICF studies using other enhanced forms in improving research subjects’ understanding [19–25], further studies on the application of the SIDCER ICF and evaluation of subjects’ understanding in a variety of study designs, settings, languages, and groups of population are required.

A closer examination of our results reveals the reasons why the participants in the enhanced ICF group did better than those in the conventional group in the post-test questionnaire. The first reason is that the enhanced ICF presents a complete set of the information required according to the first SIDCER ICF principle. Of the 13 questions in which the enhanced ICF group scored higher (Fig. 4), four items (including who can access the data, duration of the subject’s participation, expected direct/indirect benefits, and participant termination criteria) were not described in the conventional ICF (Table 1). The second reason is that the enhanced ICF uses an innovative presentation means in accordance with the third SIDCER ICF principle to convey relevant information to the target population. Nine of the 13 items (including research contact person, right to refuse, right to withdraw, subject eligibility, number of subjects required, trial procedures, foreseeable risks, anticipated expenses, and compensation for injury) were sufficiently presented in both ICFs but the enhanced ICF group was able to achieve a higher score. Interestingly, there were two items, including consequences of withdrawal and right to receive new information, which were not stated in the conventional ICF; but the proportions of the participants who correctly answered between the two groups did not show a statistically significant difference. This could be due to a practicable drawback of the evaluation tool: a close-ended question with multiple answer choices gave an individual a chance to inadvertently give the correct answer [26].

Although most of our data favored the enhanced ICF, there were two data subgroups that did not fully support our conclusion: the participants in the baby boomers generation and the participants in the educational level 3. Literature points out that each generation has a difference in perspective and perception [27]. It is possible that the presentation manners, such as figures or diagrams, used in the SIDCER ICF template might not be suitable for some generations. The increased proportion of the participants with optimal understanding by the enhanced ICF was observed in generation Y and generation X. Although there was a trend for baby boomers in the enhanced ICF group to do better, it was not statistically significantly enough to conclude that the enhanced ICF was more effective in this generation than the conventional ICF (83.3 % vs. 63.6 %, *p* = 0.105). A possible reason for this result is the small number of subjects in the baby boomers generation in comparison to the other generations (Table 2), as we did not control this factor when conducting the study. With regards to the educational level, although the result indicates that the enhanced ICF increased the proportion of the participants with optimal understanding in the participants with educational levels 1 and 2, there was little effect in the participants with educational level 3 whose understanding level was high in both groups. This is consistent with the lines of evidence demonstrating that the educational level is a major determinant to research subjects’ comprehension [28, 29]. In research practice, research subjects generally come from various backgrounds with different educational levels; thus, the SIDCER ICF could be of value.

It should be noted that this study was performed in a mock population so that the level of understanding might be either overestimated, due to the inclusion of only respondents who were willing to read a given ICF, or underestimated, owing to their limited concern for the information regarding study participation. Studies involving real research subjects of clinical studies could provide stronger evidence to support the effectiveness of the SIDCER ICF in enhancing subjects’ understanding of ICFs.

## Conclusions

Understanding of the participants in this study was improved by the enhanced ICF developed based on the SIDCER ICF principles and template. The results of this study demonstrate the potential of the application of the SIDCER ICF principles and its template in improving the quality of ICFs and subjects’ understanding in clinical research. Further studies to evaluate the applicability and effectiveness of the SIDCER ICF in a variety of clinical studies in different languages and settings are necessary.

## References

[1] Suntharalingam G, Perry MR, Ward S, Brett SJ, Castello-Cortes A, Brunner MD, Panoskaltsis N (2006). Cytokine storm in a phase 1 trial of the anti-CD28 monoclonal antibody TGN1412. N Engl J Med.

[2] Sugarman J, Levine C (2006). Risk in drug trials. Lancet.

[3] Knapp P, Raynor DK, Silcock J, Parkinson B (2009). Performance-based readability testing of participant materials for a phase I trial: TGN1412. J Med Ethics.

[4] Arora A, Rajagopalan S, Shafiq N, Pandhi P, Bhalla A, Dhibar DP, Malhotra S (2011). Development of tool for the assessment of comprehension of informed consent form in healthy volunteers participating in first-in-human studies. Contemp Clin Trials.

[5] Apseloff G, Kitzmiller JP, Tishler CL (2013). Credibility and comprehension of healthy volunteers in lengthy inpatient drug studies. Am J Ther.

[6] Fortun P, West J, Chalkley L, Shonde A, Hawkey C (2008). Recall of informed consent information by healthy volunteers in clinical trials. QJM.

[7] Fitzgerald DW, Marotte C, Verdier RI, Johnson WD, Pape JW (2002). Comprehension during informed consent in a less-developed country. Lancet.

[8] Sharp SM (2004). Consent documents for oncology trials: does anybody read these things?. Am J Clin Oncol.

[9] Berger O, Grønberg BH, Sand K, Kaasa S, Loge JH (2009). The length of consent documents in oncological trials is doubled in twenty years. Ann Oncol.

[10] Kass NE, Chaisson L, Taylor HA, Lohse J (2011). Length and complexity of US and international HIV consent forms from federal HIV network trials. J Gen Intern Med.

[11] Paasche-Orlow MK, Taylor HA, Brancati FL (2003). Readability standards for informed-consent forms as compared with actual readability. N Engl J Med.

[12] Terranova G, Ferro M, Carpeggiani C, Recchia V, Braga L, Semelka RC, Picano E (2012). Low quality and lack of clarity of current informed consent forms in cardiology: how to improve them. JACC Cardiovasc Imaging.

[13] Beardsley E, Jefford M, Mileshkin L (2007). Longer consent forms for clinical trials compromise patient understanding: so why are they lengthening?. J Clin Oncol.

[14] The Belmont Report: ethical principles and guidelines for the protection of human subjects of research (1979). U.S. Government Printing Office, Washington, D.C. 20402.

[15] Malik L, Mejia A (2014). Informed consent for phase I oncology trials: form, substance and signature. J Clin Med Res.

[16] Nishimura A, Carey J, Erwin PJ, Tilburt JC, Murad MH, McCormick JB (2013). Improving understanding in the research informed consent process: a systematic review of 54 interventions tested in randomized control trials. BMC Med Ethics.

[17] Dunn LB, Jeste DV (2001). Enhancing informed consent for research and treatment. Neuropsychopharmacology.

[18] Lancaster LC, Stillman D (2002). When generations collide.

[19] Corneli AL, Sorenson JR, Bentley ME, Henderson GE, Bowling JM, Nkhoma J, Moses A, Zulu C, Chilima J, Ahmed Y, Heilig CM, Jamieson DJ, van der Horst C, Breastfeeding Antiretroviral, and Nutrition Informed Consent Study Group (2012). Improving participant understanding of informed consent in an HIV-prevention clinical trial: a comparison of methods. AIDS Behav.

[20] Murphy DA, Hoffman D, Seage GR, Belzer M, Xu J, Durako SJ, Geiger M, Interventions ATNfHA (2007). Improving comprehension for HIV vaccine trial information among adolescents at risk of HIV. AIDS Care.

[21] Matsui K, Lie RK, Turin TC, Kita Y (2012). A randomized controlled trial of short and standard-length consent forms for a genetic cohort study: is longer better?. J Epidemiol.

[22] Paris A, Brandt C, Cornu C, Maison P, Thalamas C, Cracowski JL (2010). Informed consent document improvement does not increase patients’ comprehension in biomedical research. Br J Clin Pharmacol.

[23] Enama ME, Hu Z, Gordon I, Costner P, Ledgerwood JE, Grady C, Teams VCS (2012). Randomization to standard and concise informed consent forms: development of evidence-based consent practices. Contemp Clin Trials.

[24] Sato K, Watanabe T, Katsumata N, Sato T, Ohashi Y (2014). Satisfying the needs of Japanese cancer patients: a comparative study of detailed and standard informed consent documents. Clin Trials.

[25] Paris A, Nogueira da Gama Chaves D, Cornu C, Maison P, Salvat-Mélis M, Ribuot C, Brandt C, Bosson JL, Hommel M, Cracowski JL (2007). Improvement of the comprehension of written information given to healthy volunteers in biomedical research: a single-blind randomized controlled study. Fundam Clin Pharmacol.

[26] MacQueen KM, Chen M, Ramirez C, Nnko SE, Earp KM (2014). Comparison of closed-ended, open-ended, and perceived informed consent comprehension measures for a mock HIV prevention trial among women in Tanzania. PLoS One.

[27] Mohr NM, Moreno-Walton L, Mills AM, Brunett PH, Promes SB, Society for Academic Emergency Medicine Aging and Generational Issues in Academic Emergency Medicine Task Force (2011). Generational influences in academic emergency medicine: teaching and learning, mentoring, and technology (part I). Acad Emerg Med.

[28] Flory J, Emanuel E (2004). Interventions to improve research participants’ understanding in informed consent for research: a systematic review. JAMA.

[29] Sanchini V, Reni M, Calori G, Riva E, Reichlin M (2014). Informed consent as an ethical requirement in clinical trials: an old, but still unresolved issue. an observational study to evaluate patient’s informed consent comprehension. J Med Ethics.

